# Cracked Metal–Phenolic Networks with Durable Confinement Capillarity for Enhanced Solar Desalination

**DOI:** 10.1002/adma.202503896

**Published:** 2025-06-04

**Authors:** Zhenxing Wang, Min Hu, Lin Zhu, Jiajing Zhou, Fang He, Yanzhu Liu, Yongxiu Li, Yuexiang Li, Zhixing Lin, Frank Caruso

**Affiliations:** ^1^ School of Chemistry and Chemical Engineering Nanchang University Nanchang 330031 China; ^2^ College of Biomass Science and Engineering Key Laboratory of Leather Chemistry and Engineering of Ministry of Education National Engineering Laboratory for Clean Technology of Leather Manufacture Sichuan University Chengdu 610065 China; ^3^ Department of Chemical Engineering The University of Melbourne Parkville Victoria 3010 Australia

**Keywords:** confinement capillarity, directional crystallization, metal–organic materials, polyphenols, solar desalination

## Abstract

Solar‐driven interfacial desalination is a promising strategy to address freshwater shortages. Water evaporation can be enhanced through confinement capillarity by generating ultra‐thin water layers on the internal surfaces of porous photothermal materials. However, realizing confinement capillarity relies on coatings composed of aggregated nanospheres, which likely detach under mechanical compression, limiting their practical application. Herein, nature‐inspired crack patterns are introduced into adhesive photothermal supramolecular materials, metal–phenolic network coatings, forming C‐MPNs to achieve durable confinement capillarity. The crack patterns can be controlled to optimize water transport through narrow channels, enhancing the evaporation rate from 1.6 to 3.3 kg m^−2^ h^−1^ while preventing salt accumulation during seawater desalination. Furthermore, the cracks serve as buffer zones, significantly improving the mechanical stability of C‐MPN coatings under compression (exhibiting negligible change after 300 cycles)—overcoming a key challenge that has hindered the practical application of confinement capillarity. Furthermore, due to the enhanced confinement capillarity in C‐MPNs, high evaporation performance is sustained even as the size of the photothermal material increases—a rare characteristic among 3D photothermal materials. This work provides fundamental insights into the design of photothermal coatings with confinement capillarity, paving the way for their application in solar desalination.

## Graphical abstract

Nature‐inspired cracks are introduced into photothermal metal–phenolic networks (C‐MPNs). These crack patterns can be controlled to optimize water flow, enabling confinement capillarity to enhance the evaporation rate while preventing salt fouling. The cracks also serve as a buffer zone to significantly enhance the stability of the C‐MPNs, overcoming the bottleneck for the practical applications of confinement capillarity.

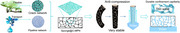

## 1 Introduction

Addressing freshwater scarcity has become a key pressing global challenge owing to population growth, climate change, and increased industrial demand.^[^ ^1^, ^2^, ^3^, ^4^ ^]^ According to the World Meteorological Organization, over 3.6 billion people endure severe water shortages for at least a month each year, a figure projected to exceed 5 billion by 2050.^[^ ^5^, ^6^ ^]^ Conventional desalination technologies, such as multistage flash evaporation, low‐temperature multi‐effect distillation systems, and reverse osmosis, have enabled significantly increased freshwater supplies; however, their reliance on fossil fuels poses substantial environmental and energy challenges.^[^ ^7^, ^8^, ^9^ ^]^ In this context, interfacial solar desalination has emerged as a promising alternative, offering a sustainable solution to addressing the global freshwater shortage.^[^ ^10^, ^11^, ^12^ ^]^ This technology harnesses abundant solar energy that is converted into heat that is directly applied to the water, thereby enhancing evaporation and condensation processes without the need for conventional nonrenewable energy source input.^[^ ^13^, ^14^, ^15^ ^]^ Significant efforts have been made to improve the performance of interfacial solar desalination systems, including designing architectures to maximize light absorption,^[^ ^16^, ^17^, ^18^ ^]^ enhancing the energy conversion efficiency of photothermal materials,^[^ ^19^, ^20^, ^21^ ^]^ and reducing the enthalpy of water evaporation.^[^ ^22^, ^23^, ^24^ ^]^ However, these approaches typically require extensive heating of the entire water body within the photothermal materials, where most of the heated water does not contribute to evaporation, thereby resulting in heat loss and limited water evaporation rates.^[^ ^25^, ^26^, ^27^, ^28^ ^]^ Therefore, advancing material design and structural engineering to optimize water flow channels in solar desalination evaporators, with the aim of minimizing thermal losses and enhancing evaporation rates, although challenging, would provide an alternative strategy.

Confinement capillarity is a ubiquitous natural phenomenon that is widely adopted to enhance water transportation efficiency across various applications.^[^ ^29^, ^30^ ^]^ This process is driven by the interplay between adhesive and cohesive forces in confined spaces and enables water movement against gravity; confinement capillarity is essential in processes ranging from plant nutrient uptake to engineered hydrodynamic systems.^[^ ^31^, ^32^, ^33^ ^]^ Leveraging confinement capillarity effects is expected to enhance the efficiency of solar desalination by optimizing water flow through narrow channels, thereby increasing the surface area for evaporation and maximizing energy utilization. For example, stacked nanospheres have been used to create water channels and induce confinement capillarity, resulting in higher water evaporation rates.^[^ ^34^ ^]^ However, significant challenges remain as aggregated nanospheres are easily displaced under mechanical compression and can detach from their substrate, thereby limiting their industrial applications.

**Scheme 1:**
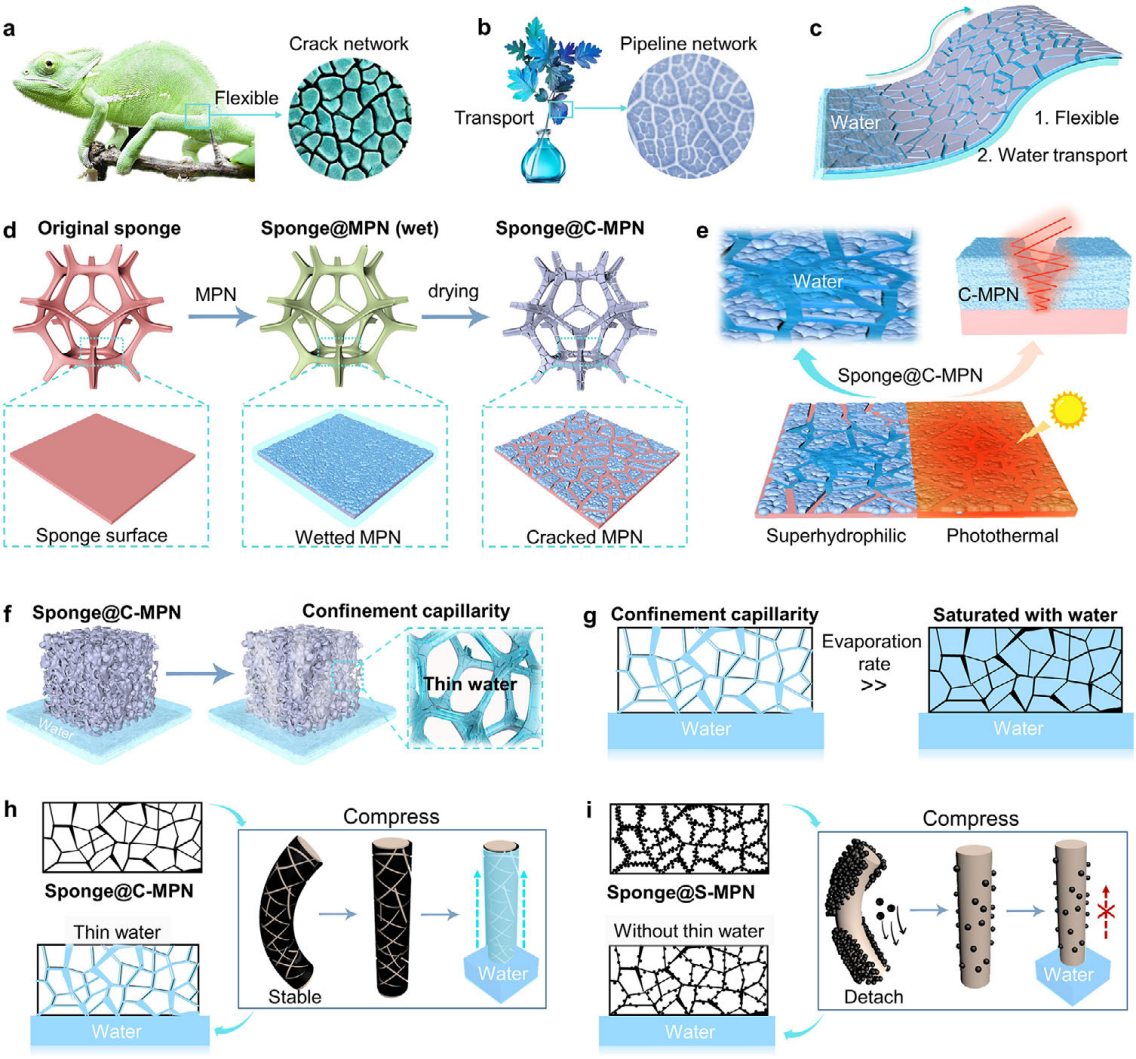
Schematic of the preparation and properties of C‐MPNs. a) Crack networks provide a chameleon skin with flexibility and resistance to bending. b) Pipeline networks in leaves ensure water distribution throughout the leaves. c) Crack networks bestow MPN coatings flexibility, bending resistance, and water transport properties. d) Schematic diagram of the preparation of a C‐MPN‐coated sponge (i.e., sponge@C‐MPN). e) Superhydrophilicity and photothermal conversion properties of C‐MPNs. f) Confinement capillarity facilitated by C‐MPNs. g) Schematic of the water distribution in photothermal materials with and without confinement capillarity. h) C‐MPNs can withstand repeated compression and maintain stable confinement capillarity for practical application. i) Detachment of S‐MPNs upon compression and consequent loss of confinement capillarity. S‐MPNs (stacked MPNs) represent the control group, consisting of MPN coatings composed of closely packed NPs without crack patterns.

Metal–phenolic networks (MPNs), composed of natural polyphenols and metal ions, are adhesive supramolecular metal–organic materials that can form coatings on various substrates within minutes, owing to their diverse interactions (e.g., hydrogen bonding, *π*–*π* stacking, and hydrophobic interactions).^[^ ^35^, ^36^, ^37^, ^38^ ^]^ Moreover, the availability of over 8000 natural functional polyphenols and a wide variety of metal ions endows MPNs with customizable and modular physicochemical properties, making them suitable for diverse applications, including biomedicine, catalysis, and solar desalination.^[^ ^19^, ^39^, ^40^, ^41^ ^]^ Herein, we introduce cracked MPNs (C‐MPNs) with photothermal and superhydrophilic properties designed to optimize water transport and evaporation in solar desalination systems, achieving long‐term stability (**Scheme** **1**). Cracks are often considered defects in material design; however, they play essential roles in nature, such as providing flexibility and resistance to bending, e.g., a chameleon skin (Scheme 1a), and facilitating water distribution across leaves (Scheme 1b). Inspired by these natural mechanisms, we engineer crack patterns as a functional feature to induce confinement capillarity in a controlled manner while buffering stress from thermal expansion and mechanical contraction (Scheme 1c). Specifically, photothermal MPNs are first coated on a commercial polyurethane sponge under optimized assembly conditions (e.g., concentration) to induce crack formation (Scheme 1d). The crack patterns (e.g., number and length) in the MPNs can be further adjusted through heat treatment to optimize their wettability and photothermal properties (Scheme 1e), which are essential for maintaining stable confinement capillarity and enhancing the evaporation rate (Scheme 1f,g). Furthermore, the C‐MPNs can endure repeated compression without sustaining damage (Scheme 1h), exhibiting significantly greater stability than the control MPN coatings. The control coatings are composed of closely packed nanoparticles (NPs) without crack patterns and are referred to as stacked MPNs (S‐MPNs, Scheme 1i). Furthermore, following an outdoor 23 day study, sponges coated with optimized C‐MPN coatings exhibit stable and high evaporation rates (2.8–3.4 kg m^−2^ h^−1^, relative humidity 32–68%, from 8:00 to 17:00 daily) when desalinating seawater (3.5 wt.%). Additionally, owing to the confinement capillarity of the C‐MPNs, the high evaporation rate is maintained even when scaling up, an outcome rarely achieved by most 3D evaporators. This strategy expands the application potential of metal–organic materials and opens an avenue through exploiting the use of cracks to improve the performance of solar desalination materials.

## 2 Results and Discussion

### 2.1 Fabrication and Characterization of C‐MPNs

**Figure 1:**
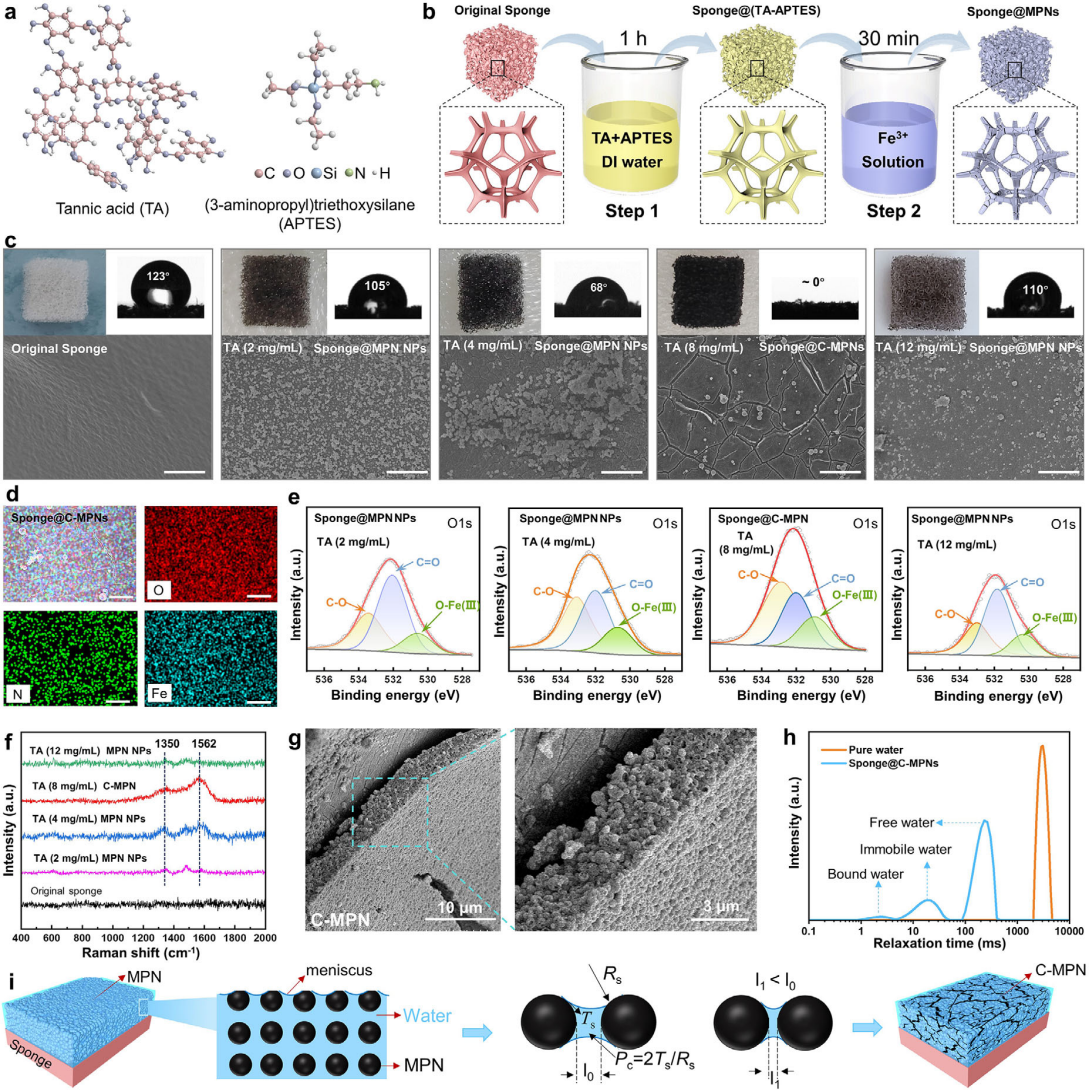
Fabrication and characterization of C‐MPNs. a) Chemical structure of TA and APTES. b) Fabrication of sponge@MPNs (sponge@MPN NPs and sponge@C‐MPNs) by a two‐step process. c) SEM images of the original sponge, sponge@MPN NPs ([TA] = 2, 4, and 12 mg mL⁻¹), and sponge@C‐MPNs ([TA] = 8 mg mL⁻¹). Scale bars are 5 µm. Insets are photographs of the corresponding sponges and their water contact angles. d) EDX mapping of sponge@C‐MPNs. Scale bars are 3 µm. e,f) Curve fitting of O1s (e) and Raman spectra (f) of the different sponges. g) SEM images of the cross‐section of a representative C‐MPN. h) Low field‐nuclear magnetic resonance (LF‐NMR) T_2_ relaxation spectrum of pure water and sponge@C‐MPNs. i) Proposed mechanism of crack formation in C‐MPNs.

Polyurethane sponges were selected as the substrates owing to their commercial availability and were coated with photothermal MPNs (**Figure** **1**a,b; Table S1, Supporting Information). Specifically, commercial polyurethane sponges were immersed in a mixture of tannic acid (TA) and (3‐aminopropyl)triethoxysilane (APTES) aqueous solution and subsequently immersed in a ferric sulfate solution to form MPN coatings on the sponges (i.e., sponge@MPNs). The physicochemical properties of the MPN coatings, including surface topography and wettability, were controlled by adjusting the precursor concentrations to facilitate the engineering of the cracks.

The formation of coatings was first visualized by a color change of the sponges from white (Figure S1, Supporting Information, photograph) to brown upon incubation with TA/APTES (Figure S2, Supporting Information, photographs) then to black upon incubation with ferric sulfate (Figure 1c, photographs, insets). Treatment with ferric sulfate solution is essential for imparting photothermal properties to the coatings by inducing black coloration while promoting cross‐linking of the TA/APTES network to enhance their stability. The morphological features of the MPN coatings prepared using varying concentrations of TA can be observed in the SEM images in Figures S3–S6 (Supporting Information). The coatings were comprised of numerous, mostly distinct, MPN NPs (100–800 nm) when a TA concentration of 2 mg mL^−1^ was used. As the precursor concentration increased, the number of NPs decreased and fused into a uniform coating until the formation of extensive crack coverage occurred at a TA concentration of 8 mg mL^−1^. These findings demonstrate the evolution of the MPNs from sparse NPs to dense coatings with cracks. When the TA concentration increased further to 12 mg mL^−1^, cracks were no longer apparent, and only sparse and limited MPN NPs were observed, resulting in a relatively light color. The coated sponges with distinct NPs as coatings are referred to sponge@MPN NPs (obtained using [TA] = 2, 4, and 12 mg mL^−1^), whereas coated sponges with cracked MPN coatings are referred to sponge@C‐MPNs (obtained using [TA] = 8 mg mL^−1^).

As the TA concentration increased, the water contact angles of the coatings decreased from 123° (original sponge) to 105° (TA = 2 mg mL^−1^), 68° (TA = 4 mg mL^−1^), and 0° (TA = 8 mg mL^−1^). The water contact angle was restored to 110° when the TA concentration increased further to 12 mg mL^−1^. The deeper the color of the coating, the higher the hydrophilicity of coated sponges, which is potentially conducive to enhancing solar evaporation performance.

High‐angle annular dark‐field and energy‐dispersive X‐ray spectroscopy (EDX) mapping revealed the presence and uniform distribution of N (originating from APTES), O, and Fe elements, confirming the formation of MPNs (Figure 1d; Figure S7, Supporting Information). As the TA concentration increased from 2 to 8 mg mL^−1^, the intensity of the band corresponding to –OH groups (hydrophilic) increased in the Fourier transform infrared (FTIR) spectra in Figure S8 (Supporting Information), whereas the ratio of C═O groups (hydrophobic) decreased in the X‐ray photoelectron spectroscopy (XPS) patterns (Figure 1e; Figure S9, Tables S2 and S3, Supporting Information). These findings are consistent with the increased hydrophilicity of the MPNs as the TA concentration increased, as discussed previously. Michael addition and Schiff base reactions between TA and APTES were not favorable in the neutral reaction solution when the TA concentration was 12 mg mL^−1^ (Figures S10 and S11, Supporting Information).^[^ ^40^ ^]^ The peaks ≈1350 and 1570 cm^−1^ in Raman spectra shown in Figure 1f were attributed to the chelation of Fe^3+^ by the oxygen atoms of the catechol groups.^[^ ^41^ ^]^ The intensity of these two peaks was the strongest when the TA concentration was 8 mg mL^−1^, suggesting strong coordination bonding within the MPN.^[^ ^19^ ^]^ This result also correlates with the high ratio of O–Fe observed in the sponge@C‐MPN (Figure 1e; Table S4, Supporting Information).

The C‐MPNs were formed by NPs according to their cross‐section scanning electron microscopy (SEM) images (Figure 1g). We speculated the presence of a significant amount of water among the NPs before drying, which was confirmed by the T_2_ relaxation time curve of the sponge@C‐MPN shown in Figure 1h. The peak at 1–10 ms represents bound water from hydrogen bond interactions between the hydrophilic C‐MPNs and water, whereas the peak at 100–10 000 ms represents free (“mobile”) water, which is primarily located on the surface of the C‐MPNs.^[^ ^42^ ^]^ Additionally, the peak at 10–100 ms represents immobilized water owing to water filling in interspaces among the NPs of the C‐MPNs. From these findings, a possible mechanism for the formation of the cracks on the sponge@C‐MPNs is proposed in Figure 1i. The interspace among the NPs of the C‐MPNs is filled with water, and a meniscus is generated between adjacent NPs during the drying process owing to the surface tension (*T*_s_).^[^ ^43^, ^44^ ^]^ This meniscus generates a capillary force (*P*_c_) that is proportional to *T*_s_ but inversely proportional to the radius of curvature (*R*_s_) of the meniscus.^[^ ^45^ ^]^ As a combined result of *T*_s_ and *P*_c_, the NPs are drawn close together, reducing the aperture (*l*) and leading to shrinkage and cracking of the MPN coating.

### 2.2 Effect of Crack Structure on Evaporation Performance

**Figure 2:**
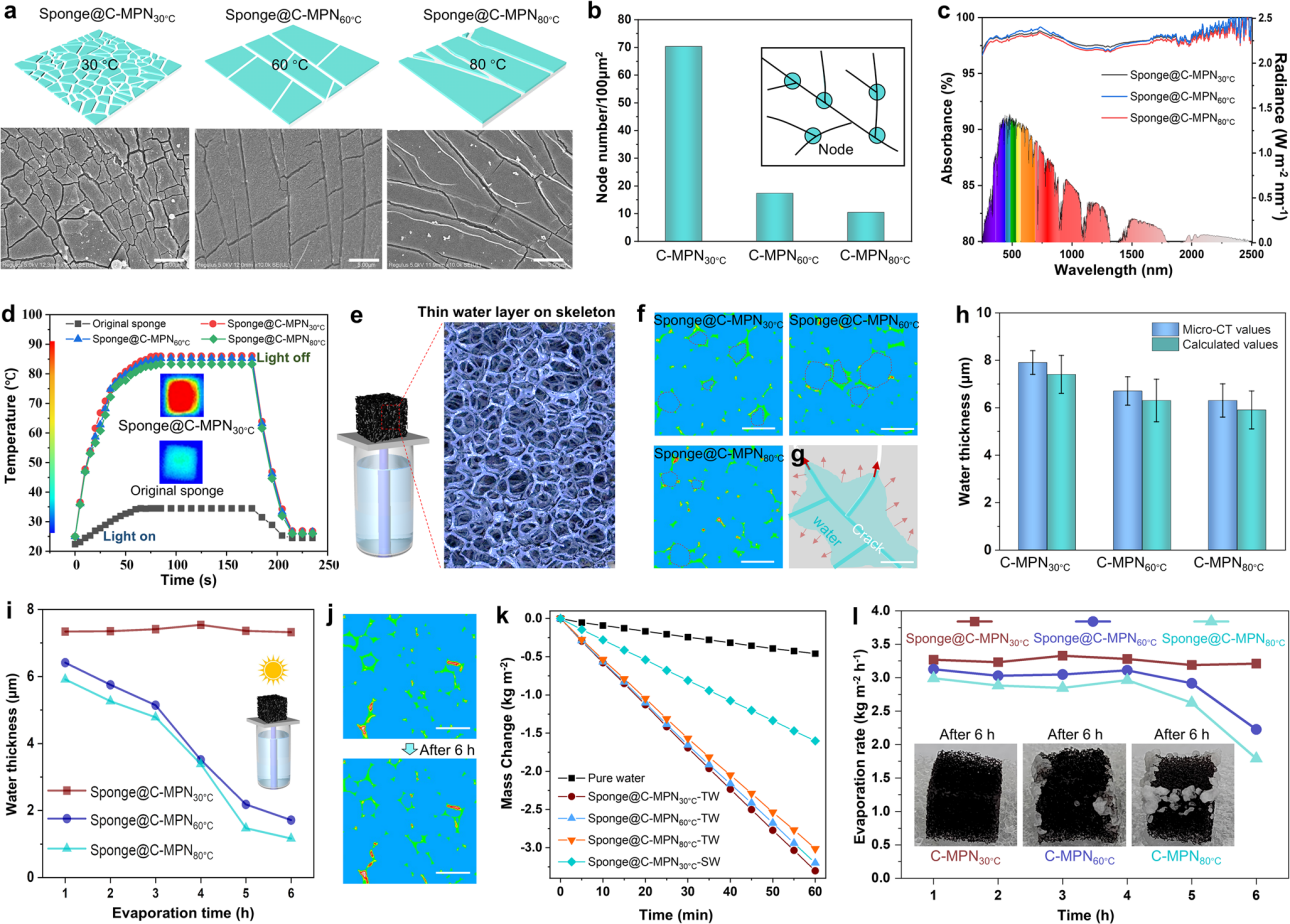
Effect of cracks on desalination performance. a) SEM images of cracks formed at different drying temperatures (30, 60, and 80 °C). Scale bars are 2 µm. b) Node numbers and c) UV–vis absorption spectra of C‐MPNs with different cracks obtained at different drying temperatures. d) Thermal infrared images and related temperature change of different sponge@C‐MPNs upon exposure to one sun irradiation. e) 3D Micro‐CT image showing a thin water layer on the C‐MPN. f) High‐resolution micro‐CT images of sponge@C‐MPNs with different cracks. Scale bars are 100 µm. g) Schematic showing the formation of a thin water layer on the skeleton of sponge@C‐MPN. h) Thickness of the water layer on the skeleton of different sponges. Data are shown as the mean ± standard deviation (SD, *n* = 4). i) Changes in water layer thickness of different sponges during evaporation. j) Micro‐CT images of sponge@C‐MPN_30°C_ before and after evaporation under one sun irradiation for 6 h. k) Changes in the amount of water evaporated as a function of time using evaporators under one sun irradiation. l) Evaporation rate changes in different sponges during treatment of 3.5 wt.% NaCl solution under one sun irradiation for 6 h. Insets are photographs of the sponges after desalination.

The crack architecture in C‐MPNs can be tailored by adjusting the drying temperature (**Figure** **2**a). For instance, a reticulate crack structure formed on sponge@C‐MPN using a drying temperature of 30 °C (sponge@C‐MPN_30°C_, Figure 2a; Figure S12, Supporting Information) (the coatings discussed previously were formed using a drying temperature of 30 °C). Increasing the drying temperature to 60 °C resulted in the formation of quasi‐parallel long cracks interspersed with short cracks (Figure 2a; Figure S13, Supporting Information). At 80 °C, long cracks remained, unlike the short cracks connecting adjacent long cracks, that were absent (Figure 2a; Figure S14, Supporting Information). We then quantified the crack structure using node numbers, where a node is defined as an intersection between cracks (Figure 2b). Sponge@C‐MPN_30°C_ featured a node number of 70.3 per 100 µm^2^, which is ≈4 times and 7 times higher than those of sponge@C‐MPN_60°C_ (17.3 per 100 µm^2^) and sponge@C‐MPN_80°C_ (10.4 per 100 µm^2^).

The effect of crack structures on light absorption and photothermal performance of the coated sponges was also investigated. As shown in Figure 2c, all sponges (obtained following drying at 30, 60, and 80 °C) exhibited a high degree of light absorption (>95%), with sponge@C‐MPN_30°C_ displaying a slightly higher degree of light absorption than sponge@C‐MPN_60°C_ and sponge@C‐MPN_80°C_. Given that the compositions of the C‐MPNs are identical, the enhanced light absorption of sponge@C‐MPN_30°C_ is likely due to its denser crack network, which enhances light absorption through multiple reflections and scattering.^[^ ^46^ ^]^ The light‐to‐thermal conversion performance of the sponges correlated with their light absorption properties. Under one sun irradiation, the surface temperature of sponge@C‐MPN_30°C_ (86 °C) was comparable to those of sponge@C‐MPN_60°C_ (85.2 °C) and sponge@C‐MPN_80°C_ (83.3 °C), but significantly higher than that of the original sponge (34.5 °C), due to the photothermal effects conferred by the cracked MPNs (Figure 2d). Notably, these photothermal properties primarily result from the composition‐induced black coloration and are comparable to those of the most advanced photothermal materials; moreover, our materials are prepared via a facile process (i.e., aqueous synthesis without the need for high pressure or temperature).^[^ ^47^, ^48^, ^49^, ^50^, ^51^, ^52^, ^53^ ^]^

The confinement capillarity of the different sponge@C‐MPNs was characterized using micro‐computed tomography (micro‐CT) after being irradiated under one sun for 15 min (Figure 2e; Figure S15, Supporting Information). The 3D micro‐CT image revealed abundant water layers (as indicated by the bright areas) on the sponge skeleton, whereas no water was observed within the pores of the sponge (Figure 2e). The distribution of these water layers was detailed further using 2D micro‐CT imaging. In Figure 2f, the cross‐section of the sponge skeleton was indicated by the red dashed lines, and the water layers were highlighted in green. These images confirm that water predominantly resides within the cracks rather than within the pores of the sponge, indicating that the confinement capillarity (which is facilitated by the cracks and superhydrophilicity) generates sufficient capillary force to transport water (Figure 2g). According to the micro‐CT analysis, the thicknesses of the water layers on sponge@C‐MPN_30°C_, sponge@C‐MPN_60°C_, and sponge@C‐MPN_80°C_ were ≈7.9 ± 0.5 µm, 6.7 ± 0.6 µm, and 6.3 ± 0.7 µm, respectively (Figure 2h). Additionally, these thicknesses closely match the theoretically calculated values. The calculated thickness values were used to investigate the change in water thickness of the sponges during a continuous 6 h solar evaporation process. As shown in Figure 2i, under one sun irradiation for 6 h, the water thickness of sponge@C‐MPN_30°C_ remained stable (7.3 µm), whereas the water thickness gradually decreased to 1.7 µm for sponge@C‐MPN_60°C_ and to 1.2 µm for sponge@C‐MPN_80°C_. Furthermore, no apparent change in the water layer (green color) was observed for sponge@C‐MPN_30°C_ following the solar evaporation experiment, as characterized by micro‐CT (Figure 2j). These results indicate that the cracks on sponge@C‐MPN_30°C_ provide sufficient confinement capillarity to maintain the water thickness for prolonged evaporation, outperforming the cracks within sponge@C‐MPN_60°C_ and sponge@C‐MPN_80°C_.

The water evaporation rates of the different sponge@C‐MPNs were further investigated. As observed in Figure 2k, the water evaporation rate (under one sun irradiation) of sponge@C‐MPN_30°C_ with a thin water (TW) layer (referred to as sponge@C‐MPN_30°C_‐TW) was as high as 3.3 kg m^−2^ h^−1^, which was slightly higher than that for the sponge@C‐MPN_60°C_‐TW (3.2 kg m^−2^ h^−1^) and the sponge@C‐MPN_80°C_‐TW (3.0 kg m^−2^ h^−1^), and twice higher than that for the sponge@C‐MPN_30°C_ saturated with water (SW) (referred to as sponge@MPN_30°C_‐SW) (1.6 kg m^−2^ h^−1^). These findings demonstrate that the thin water layer realized by the C‐MPNs via confinement capillarity can significantly improve the water evaporation rate. Notably, all sponges with confinement capillarity exhibited a high degree of water evaporation rate (>87%, Figures S16 and S17, Supporting Information). Additionally, the continuous evaporation performance of 3.5% NaCl solution for 6 h under one sun irradiation was evaluated. As shown in Figure 2l, the evaporation rate of sponge@C‐MPN_30°C_ remained ≈3.2 kg m^−2^ h^−1^ throughout the 6 h evaporation process, and no salt crystals appeared on the sponge surface (Figure S18, Supporting Information). This is likely due to a stable water supply maintaining a consistent water layer thickness (i.e., ≈7.9 µm, Figure 2i), which serves as a buffer zone to dissolve salts and inhibit their crystallization. In contrast, the evaporation rates of sponge@C‐MPN_60°C_ and sponge@C‐MPN_80°C_ decreased by 29% (from 3.1 to 2.2 kg m^−2^ h^−1^) and 38% (from 2.9 to 1.8 kg m^−2^ h^−1^) after 6 h of irradiation, respectively, with abundant salt crystals appearing on both sponges. This can be attributed to the significant reduction in the water layer thickness during evaporation (Figure 2i), which leads to inadequate water replenishment and the inability to dissolve accumulating salts, ultimately resulting in salt crystallization. The high and stable evaporation rate of the sponge@C‐MPN_30°C_ toward brine indicates that the cracks in the sponge can better balance the water supply and water evaporation. Therefore, sponge@C‐MPN_30°C_ was selected for the subsequent experiments.

### 2.3 Compression Resistance of Sponge@C‐MPN_30°C_

**Figure 3:**
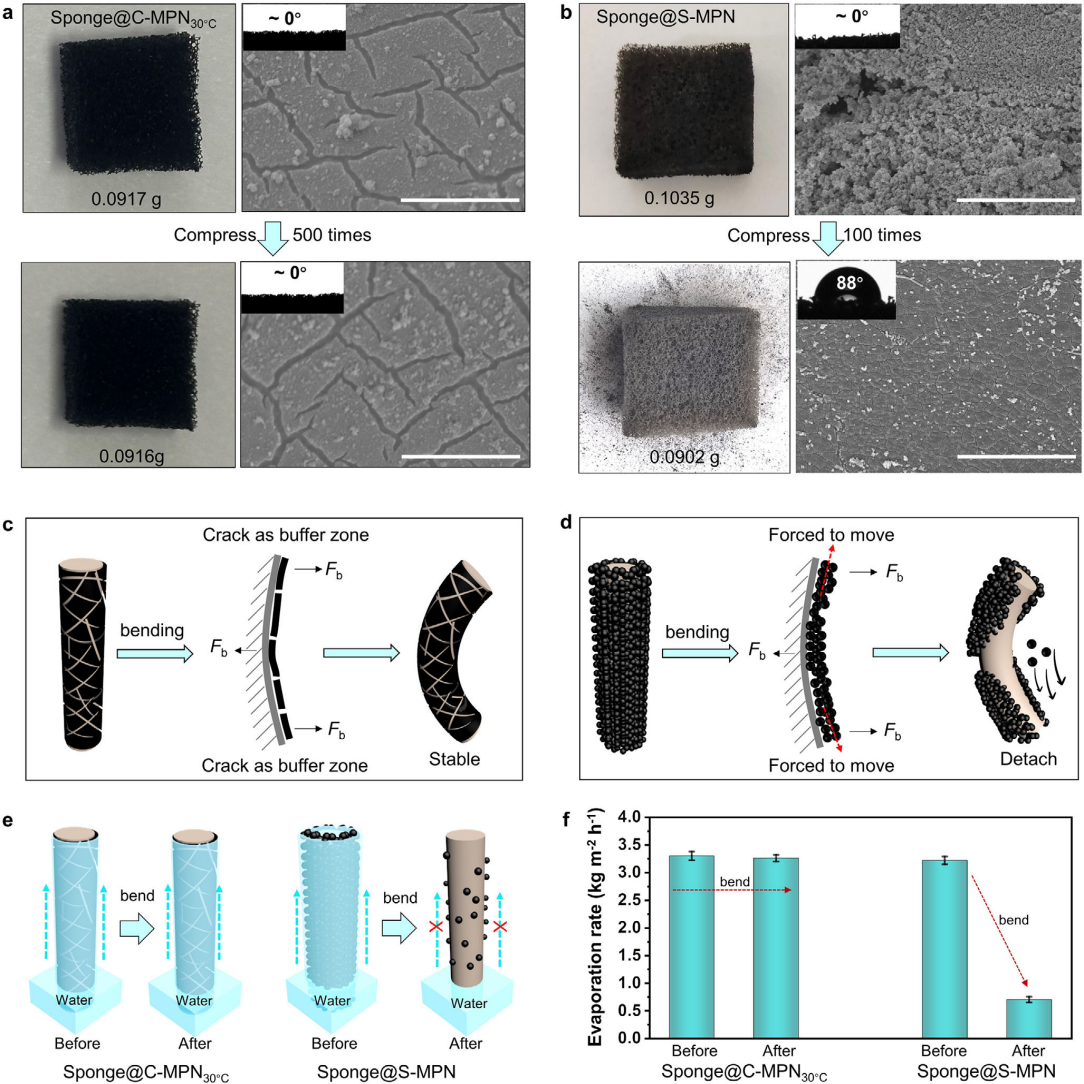
Compression resistance of C‐MPN_30°C_. a,b) Photographs and SEM images of sponge coated with C‐MPN_30°C_ (a) or sponge@S‐MPN (b) before and after compression for 500 or 100 cycles using a 20 g weight. Scale bars are 10 µm. c,d) Schematic diagrams showing the stability of C‐MPN_30°C_ (c) and reduced stability of sponge@S‐MPN (d) before and after compression. e) Schematic diagram showing the stable confinement capillarity of sponge@C‐MPN_30°C_ and the loss of confinement capillarity of sponge@S‐MPN after compression. f) Water evaporation rate of sponge@C‐MPN_30°C_ and sponge@S‐MPN before and after compression. Data are shown as the mean ± SD (*n* = 4).

The stability of the C‐MPNs upon compression or bending is essential for their practical application. As shown in **Figure** **3**a, sponge@C‐MPN_30°C_ displayed a negligible change in weight (from 0.0917 to 0.0916 g) after 500 compression cycles (Figure S19, Movies S1 and S2, Supporting Information). In addition, the integrity and superhydrophilicity of the C‐MPNs on the sponge surface were well preserved. In contrast, a sponge with typical coatings formed by stacked MPN NPs (i.e., sponge@S‐MPNs)^[^ ^34^ ^]^ deteriorated significantly when subjected to similar conditions (Figure 3b; Figure S20, Supporting Information). After 100 compression cycles, many nanospheres detached from the sponge, as indicated by a weight reduction from 0.1035 to 0.0902 g and color lightening of the sponge. Additionally, the superhydrophilicity of the sponge@S‐MPNs significantly decreased, with the water contact angle increasing from 0 to 88°, resulting in a substantial loss of capillary confinement. This was further confirmed by SEM, wherein the sponge featured a relatively smooth surface (Figure 3b; Figure S21, Supporting Information). The marked differences between these two sponges under compression are schematically illustrated in Figure 3c,d. For sponge@C‐MPN_30°C_, the crack structure acts as a buffer against compression forces (Figure 3c), thus preserving the integrity of the coating. Conversely, the closely agglomerated MPN nanospheres, which lack buffer zones, are displaced across the sponge surface during compression, leading to their detachment (Figure 3d). Consequently, sponge@C‐MPN_30°C_ retained its confinement capillarity after compression unlike sponge@S‐MPNs (Figure 3e). The water evaporation rate of sponge@C‐MPN_30°C_ remained stable at 3.3 kg m^−2^ h^−1^, whereas that of the sponge@S‐MPNs decreased considerably from 3.2 to 0.7 kg m^−2^ h^−1^ after compression for 100 times (Figure 3f). The findings infer that the C‐MPNs developed in this work offer substantial advantages over agglomerated NPs for practical application. Additionally, sponge@C‐MPN_30°C_ demonstrated acid–base resistance (pH = 3–11) and stability against vigorous rinsing (Figure S22, Supporting Information).

### 2.4 Scaling Up and Outdoor Performance

**Figure 4:**
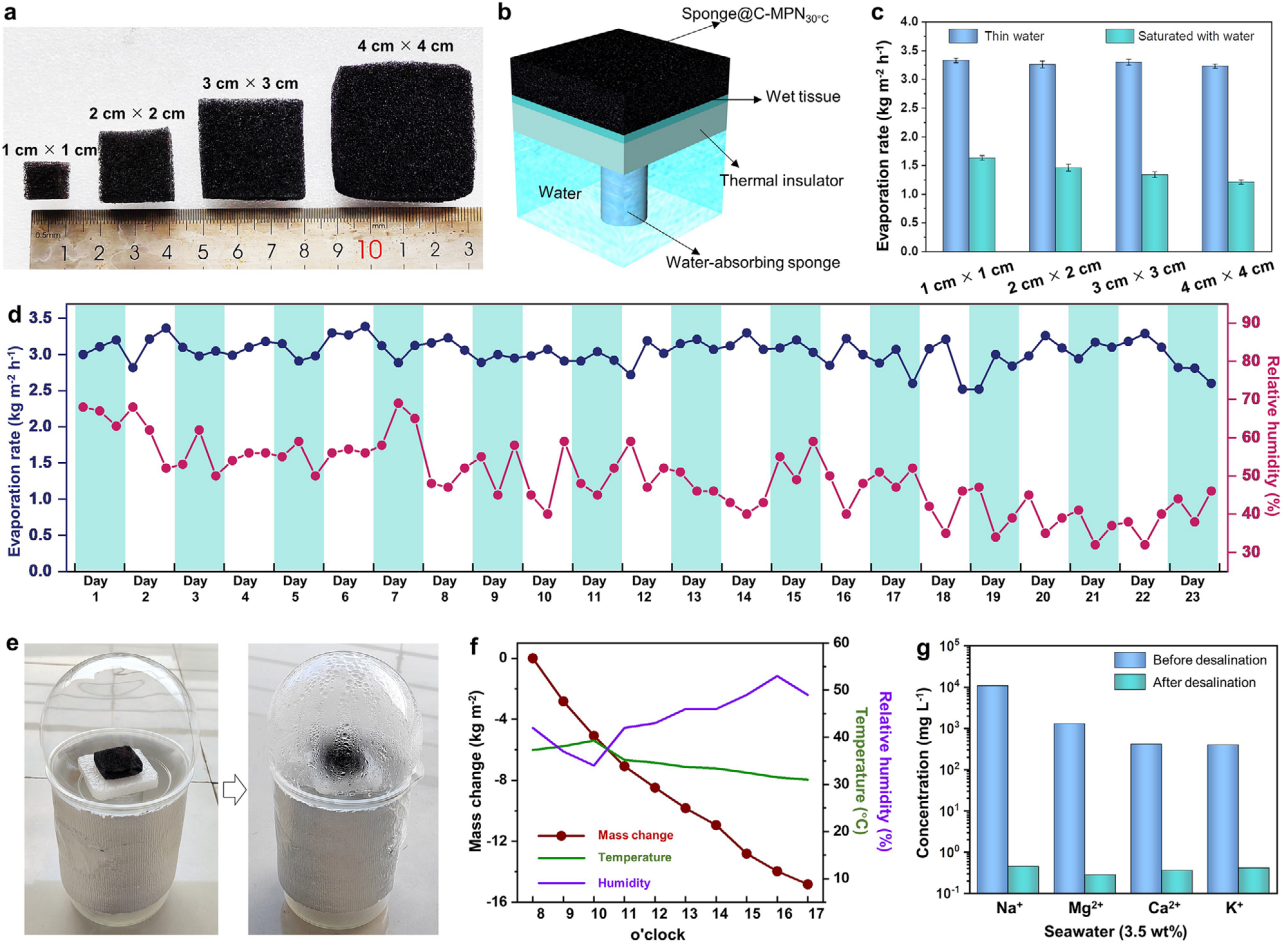
Size effect and outdoor performance of C‐MPN_30°C_. a) Photograph of sponge@C‐MPN_30°C_ of different dimensions. b) Schematic diagram of the water supply device used for sponge@C‐MPN_30°C_. c) Water layer thickness and evaporation rate of sponge@C‐MPN_30°C_ of varying dimensions. Data are shown as the mean ± SD (*n* = 4). d) Long‐term evaporation performance of sponge@C‐MPN_30°C_ for seawater. e) Photographs of an evaporator device based on sponge@C‐MPN_30°C_ before (left) and after (right) outdoor seawater desalination. f) Outdoor experimental data of water evaporation with varying ambient temperatures and humidity levels. g) Concentrations of different metal ions before and after desalination of seawater.

3D evaporators have attracted considerable attention for their ability to enhance water evaporation rates through side‐area effects, which effectively increase the evaporation surface and harness energy from the surrounding environment. However, maintaining high water evaporation rates during scaling up for industrial applications is typically challenging owing to the significant reduction in side‐area effects (i.e., a decrease in the ratio of the side surface area to the top surface area).^[^ ^10^ ^]^ To address this issue, we exploited the confinement capillarity of C‐MPN coatings. As proof of concept, four sponge@C‐MPN_30°C_ of different dimensions (length × width = 1 cm × 1 cm; 2 cm × 2 cm; 3 cm × 3 cm; and 4 cm × 4 cm; the thickness was fixed at 1 cm) were prepared (**Figure** **4**a) with different water saturation states. A thin commercial wet tissue was used in a water supply device to ensure uniform water supply coverage below the whole surface of the sponge (Figure 4b). The average water layer thickness of the four sponge@C‐MPN_30°C_ was comparable and corresponded to a thin water layer (Figure S23, Supporting Information), demonstrating that the confinement capillarity was consistently achievable across the different sponges. The corresponding evaporation rates were 3.33 ± 0.04, 3.26 ± 0.06, 3.30 ± 0.05, and 3.23 ± 0.04 kg m^−2^ h^−1^ (Figure 4c), indicating that high performance can be maintained during scaling up. For comparison, when the 3D evaporators were fully saturated with water, the corresponding evaporation rate decreased from 1.63 kg m^−2^ h^−1^ (1 cm × 1 cm) to 1.21 kg m^−2^ h^−1^ (4 cm × 4 cm) (Figure 4c). These results demonstrate the advantages of C‐MPNs with confinement capillarity for practical application.

Furthermore, sponge@C‐MPN_30°C_ displayed long‐term stability in outdoor seawater desalination (3.5 wt.%). As shown in Figure 4d, sponge@C‐MPN_30°C_ was irradiated for 9 h (outdoors, from 8:00 to 17:00) over a 23‐day period. Throughout this period, the water evaporation rate consistently ranged between 2.8 and 3.4 kg m^−2^ h^−1^, with superhydrophilicity maintained after 23 days of operation (Figure S24 and Movie S3, Supporting Information), despite fluctuating relative humidity levels (32–68%). The outdoor desalination performance of sponge@C‐MPN_30°C_ toward seawater was further highlighted (Figure 4e,f) as the device produced 14 kg of clean water per square meter over 9 h (from 8:00 to 17:00) and significantly reduced the concentrations of Na^+^, K^+^, Mg^2+^, and Ca^2+^ in the treated water by 3–4 orders of magnitude (Figure 4g), thus meeting the World Health Organization standard for drinking water.^[^ ^54^ ^]^

## 3 Conclusion

We have introduced and engineered cracks, which are typically considered flaws, into photothermal coatings (i.e., C‐MPNs), which not only significantly enhance solar desalination performance through confinement capillarity but also exhibit excellent compression resistance. Additionally, the C‐MPNs enable long‐term stable seawater evaporation at high evaporation rates, which is maintained when scaling up. This work is expected to advance both fundamental and applied research in the rational design of photothermal coatings for solar desalination. By integrating material design and morphology control, this study broadens the potential application of cracked structures toward the development of advanced functional coatings.

## Conflict of Interest

The authors declare no conflict of interest.

## Supporting information



Supporting Information

Supplemental Movie 1

Supplemental Movie 2

Supplemental Movie 3

## Data Availability

The data that support the findings of this study are available from the corresponding author upon reasonable request.
