# Supplementary material for: Cracked Metal–Phenolic Networks with Durable Confinement Capillarity for Enhanced Solar Desalination
Source: Adv Mater. 2025 Jun 4;37(33):2503896. doi: 10.1002/adma.202503896 (PMC12369692; doi:10.1002/adma.202503896)
Supplement: Supplementary file 1 — Supporting Information [file ADMA-37-2503896-s002.pdf]

ADVANCED MATERIALS

Supporting Information

for *Adv. Mater.*, DOI 10.1002/adma.202503896

Cracked Metal–Phenolic Networks with Durable Confinement Capillarity for Enhanced Solar Desalination

Zhenxing Wang, Min Hu, Lin Zhu, Jiajing Zhou, Fang He, Yanzhu Liu, Yongxiu Li, Yuexiang Li, Zhixing Lin* and Frank Caruso**

Supporting Information

Cracked Metal–Phenolic Networks with Durable Confinement Capillarity for Enhanced Solar Desalination

*Zhenxing Wang,*** *Min Hu, Lin Zhu, Jiajing Zhou, Fang He, Yanzhu Liu, Yongxiu Li, Yuexiang Li, Zhixing Lin,* and Frank Caruso***

Experimental Section

Materials

Tannic acid (TA), ferric sulfate, tris(hydroxymethyl)methylaminomethane (Tris, 99%), (3-aminopropyl)triethoxysilane (APTES), ethanol, and NaCl (99%) were obtained from Aladdin (China). Commercial polyurethane sponges were supplied by a local store. Seawater was obtained from South China Sea. All chemicals were used as received.

Preparation of sponge@C-MPNs and sponge@MPN NPs

For the synthesis of metal–phenolic networks with cracked patterns coated on sponge (i.e., sponge@MPNs), a known amount of TA (20, 40, 80, or 120 mg) was first dissolved in deionized water (10 mL) to generate TA solutions of varying concentrations of 2, 4, 8, 10, and 12 mg mL^{-1} to which APTES (80 mg) was added. Then, a piece of polyurethane sponge (1 cm × 1 cm × 1 cm), which was previously washed with ethanol, was immersed into the above mixture and shaken at room temperature for 1 h. The sponge was then retrieved and washed with ethanol and deionized water before immersion into an aqueous solution of ferric sulfate (2 mg mL^{-1}) for 30 min. Then, the sponge was retrieved, rinsed thoroughly with deionized water, and stored in water before use. The preparation conditions used for the preparations of the different modified sponges are shown in Table S1. Among the different sponges obtained, only sponge@MPN prepared using a TA concentration of 8 mg mL^{-1} featured cracks and is referred to as sponge@C-MPN. Modified sponges obtained using other TA concentrations are denoted as sponge@MPN nanoparticles (NPs).

Sponge@C-MPN was compressed between two pieces of glass to discharge water filled in the large pores of the sponge. To generate different cracks, the sponge was dried at 30, 60, and 80 °C for 6 h in an oven. The resultant sponges with different cracks generated at different drying temperatures are termed as sponge@C-$MPN_{30℃}$, sponge@C-$MPN_{60℃}$, and sponge@C-$MPN_{80℃}$.

Preparation of sponge@S-MPNs

For the synthesis of MPN coatings, composed of closely packed NPs without crack patterns, on sponges and referred to as stacked MPN-coated sponge (i.e., sponge@S-MPNs), TA (50 mg) was dissolved in Tris-HCl buffer solution (25 mL, pH = 8.5) to which ethanol (5 mL) containing APTES (75 mg) was added. A piece of polyurethane sponge (1 cm × 1 cm × 1 cm), which was previously washed with ethanol, was added to the mixed solution and shaken at room

temperature for 12 h. The modified sponge was then thoroughly washed with deionized water to remove impurities from the surface. Finally, the sponge was immersed in ferric sulfate solution (2 mg mL^{-1}) for 2 h to obtain the sponge@S-MPNs.

Preparation of sponge@C-MPN-SW and sponge@C-MPN-TW

sponge@C-MPN-SW (saturated with water) was obtained following immersion of sponge@C-MPN in deionized water and retrieval without any compression, resulting in a sponge that was completely filled (saturated) with water.

sponge@C-MPN was immersed in deionized water or seawater and then retrieved. Then, the sponge was squeezed tightly between two pieces of glass to discharge water from the large pores of the sponge and subsequently released to generate a thin water (TW) layer on the skeleton of the sponge. The resultant sponge with a thin water layer is referred to as sponge@C-MPN-TW. Notably, the squeezing process facilitates the formation of confinement capillarity via the thin water layer within seconds, whereas sponges without the squeezing or compression step can also achieve this effect over a longer time (i.e., several minutes).

Measurement of water layer thickness and distribution

The thickness and spatial distribution of the water layer on sponge@C-MPNs were characterized using micro-computed tomography (micro-CT, ZEISS Xradia 620 Versa). To prepare the sample, sponge@C-MPN (1 cm × 1 cm × 1 cm) was immersed in 10 wt% NaI solution and then removed. The sponge was then immediately compressed between two glass slides to expel water from the macropores and subsequently released, allowing a thin water film to form along the sponge skeleton. The treated sample was then mounted on a water supply device and subjected to micro-CT imaging. The thickness of the water layer was calculated by the equation: $Th_w = w_w/s_{sp}$, where Th_w is the thickness of the water layer on the sponge, w_w is the weight of water within the sponge, and s_{sp} is the surface area of the sponge.

Low-field nuclear magnetic resonance (LF-NMR) relaxation measurements were performed using a PQ001 LF-NMR analyzer (Niumag Electric Co., Shanghai, China), operating at a magnetic field strength of 0.5 T with a spectrometer frequency of 23 MHz under ambient conditions. Samples were placed in 40-mm glass tubes and inserted into the NMR probe. T_2 relaxation times were acquired using the Carr–Purcell–Meiboom–Gill (CPMG) sequences, with an inter-echo time (τ) of 250 μs. A total of 3000 echoes were collected over 16 scans. The

resulting data were analyzed using MultiExp Inv Analysis software (Niumag Electric Corporation, Shanghai, China). The analysis revealed three distinct T_2 relaxation water populations—bound water, immobilized water, and free water—identified via the cumulative integration method.

Stability of sponge@C-MPN and sponge@S-MPN

To evaluate the stability of the sponges, sponge@C-MPN$_{30°C}$ and sponge@S-MPN were compressed with a weight of 20 g for 300 and 100 times, respectively. The weight, surface topography, and evaporation performance of the sponges before and after compression were investigated. In addition, the water evaporation performances of sponge@C-MPN$_{30°C}$ before and after rinsing (1000 rpm) and acid and alkali treatment (pH = 3–11) were investigated.

Indoor solar-driven water evaporation experiments

The indoor water evaporation performance of the sponges was investigated under simulated solar conditions using a Xenon lamp as the light source (MC-XS500, Beijing Merry Change Technology Co., Ltd.); the simulated output solar flux was 1 kW m^{-2} at room temperature. A light flux intensity meter was used to measure the solar flux (MC-PM100C, Beijing Merry Change Technology Co., Ltd.). A sponge@C-MPN was attached to the device shown in Figure S15 for the evaporation experiments, and the mass of the water loss was recorded using an electronic balance. All evaporation rates were measured after stabilization under one sun for 15 min.

The evaporation efficiency (η_e) was calculated using the following equation:

$$\eta_e = \dot{m}h/(C_{opt}P_0) \quad (1)$$

where $\dot{m}$ is net evaporation rate (i.e., the measured rate minus the rate attributed to side surface evaporation under illumination), P_0 is the solar irradiation power (1 kW m^{-2} in this study), C_{opt} refers to the optical concentration, and h is the evaporation enthalpy of water (2450 kJ kg^{-1}).

Outdoor long-term solar-driven seawater evaporation experiments

The device with sponge@C-MPN$_{30°C}$ was irradiated daily under the sun for 8 h (from 8:00 to 17:00) for 23 days. Seawater from the South China Sea was used for the evaporation rate experiments. The temperature, relative humidity, and mass of water loss were recorded every 3 h. The purified water was collected for inductively coupled plasma mass spectrometry analysis.

Characterization

Scanning electron microscopy (SEM; S-4500, Hitachi, Japan) was used to examine the morphologies of the sponges. The chemical groups and elemental composition of the sponges were determined by attenuated total reflectance Fourier transform infrared (FTIR) spectroscopy (Spectrum GX, PerkinElmer, USA), X-ray photoelectron spectroscopy (XPS; PHI-5700, PerkinElmer, USA), and SEM/energy-dispersive X-ray (EDX) spectroscopy (S-4500, Hitachi, Japan). The reflectance (*R*) and transmittance (*T*) spectra of the sponges were recorded on a U-4100 (Hitachi), and the absorbance (*A*) spectra was obtained by $A = 1 - T - R$. The temperature and infrared images of the sponges were recorded on a thermal infrared imager (Fluke Tis10). The wettability of the sponges was examined by a contact angle measuring system (SL200KB, KINO, China). The concentrations of metal ions were determined by inductively coupled plasma mass spectrometry (Agilent 7500ce, USA).

Supporting Figures, Tables, and Movies

Table S1. Preparation conditions of different sponge@MPNs

Sponge	Step 1			Step 2	
	TA [mg mL^{-1}]	APTES [mg mL^{-1}]	Reaction time [h]	Fe^{3+} [mg mL^{-1}]	Reaction time [h]
sponge@MPN NPs	2	8	1	2	0.5
sponge@MPN NPs	4	8	1	2	0.5
sponge@C-MPN	8	8	1	2	0.5
sponge@MPN NPs	12	8	1	2	0.5

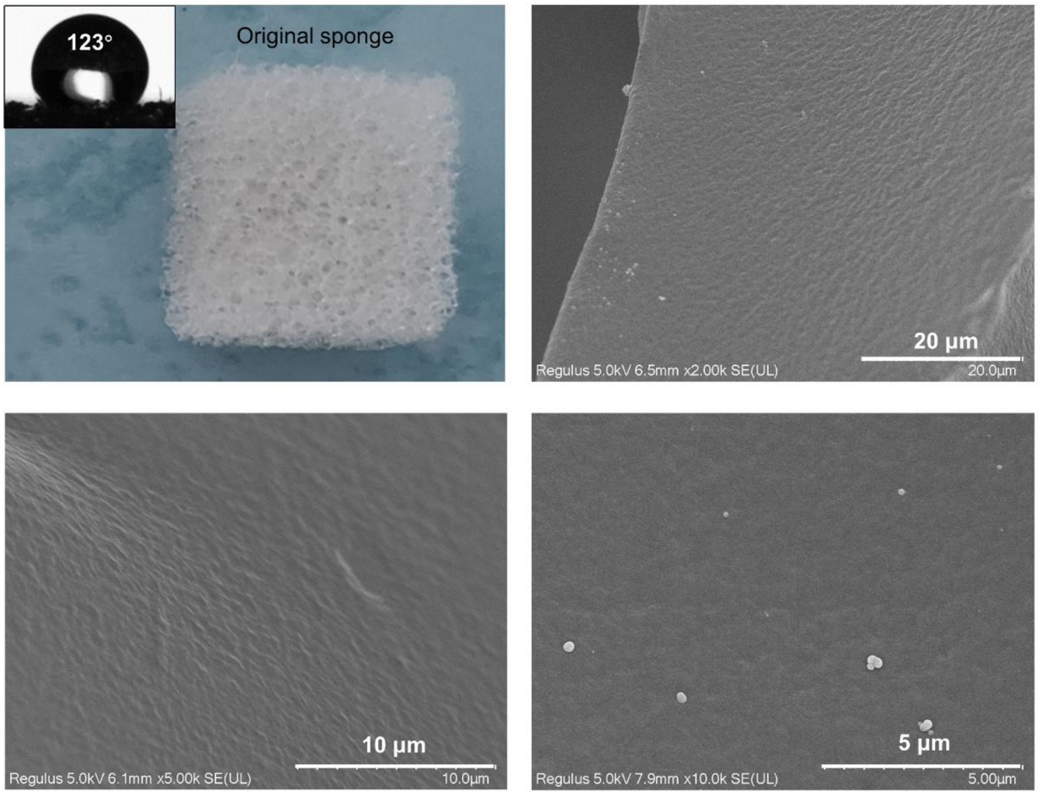

Figure S1. Photograph and SEM images of the original sponge and the corresponding water contact angle (inset).

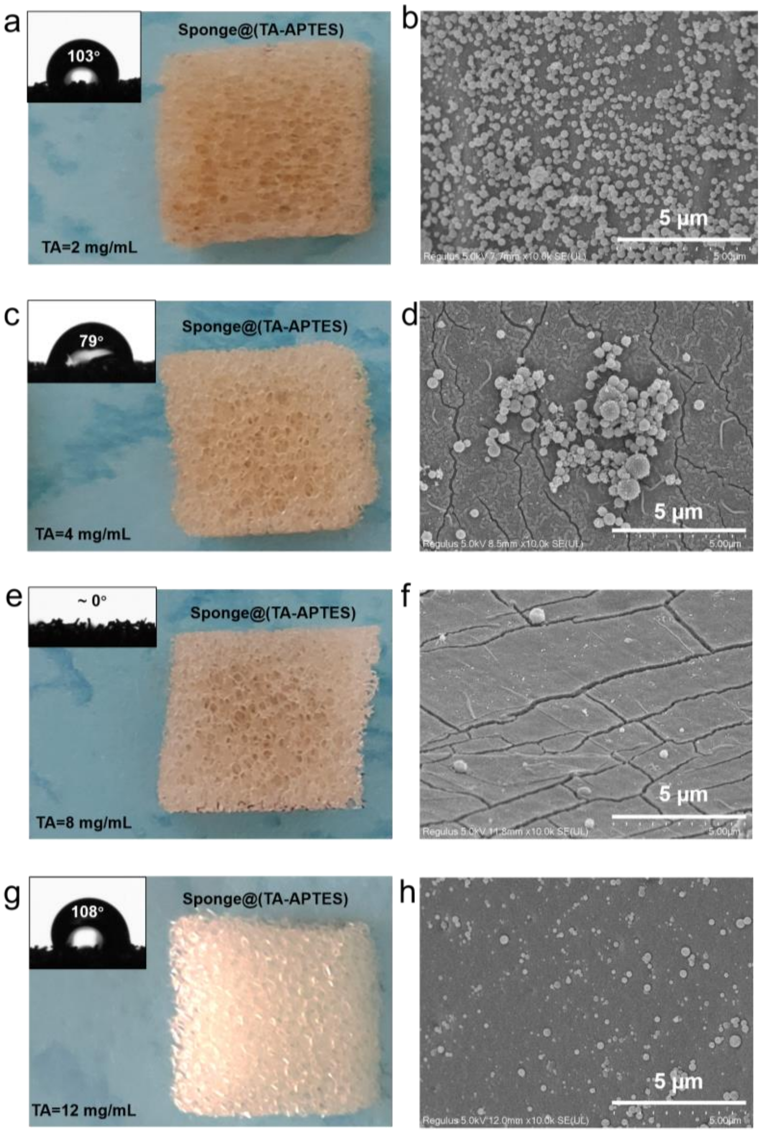

Figure S2. (a–h) Photographs and corresponding water contact angles (insets) (a, c, e, g) and corresponding SEM images (b, d, f, h) of sponges coated with TA-APTES at varied TA concentrations: 2 mg mL^{-1} (a, b), 4 mg mL^{-1} (c, d), 8 mg mL^{-1} (e, f), and 12 mg mL^{-1} (g, h).

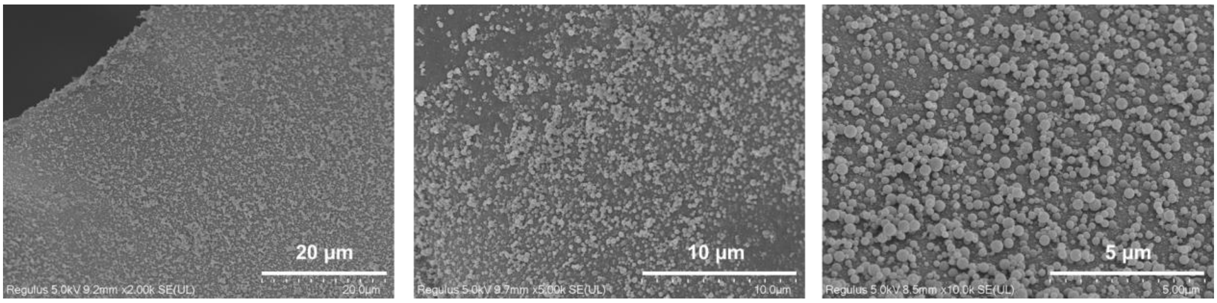

Figure S3. SEM images of sponge@MPN NPs ([TA] = 2 mg mL^{-1}) (obtained after incubation of sponge@TA-APTES with ferric sulfate).

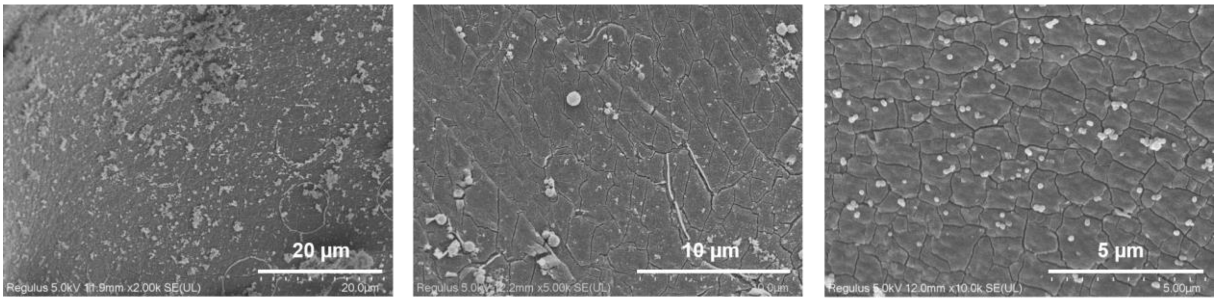

Figure S4. SEM images of sponge@MPN NPs ([TA] = 4 mg mL^{-1}) (obtained after incubation of sponge@TA-APTES with ferric sulfate).

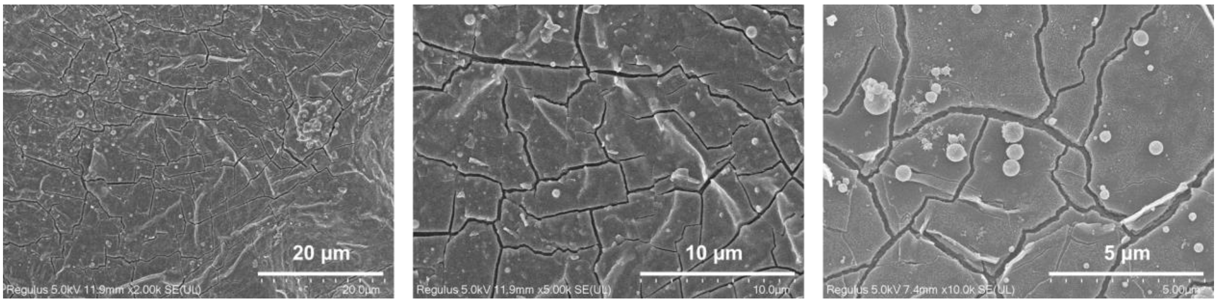

Figure S5. SEM images of sponge@C-MPNs ([TA] = 8 mg mL^{-1}) (obtained after incubation of sponge@TA-APTES with ferric sulfate).

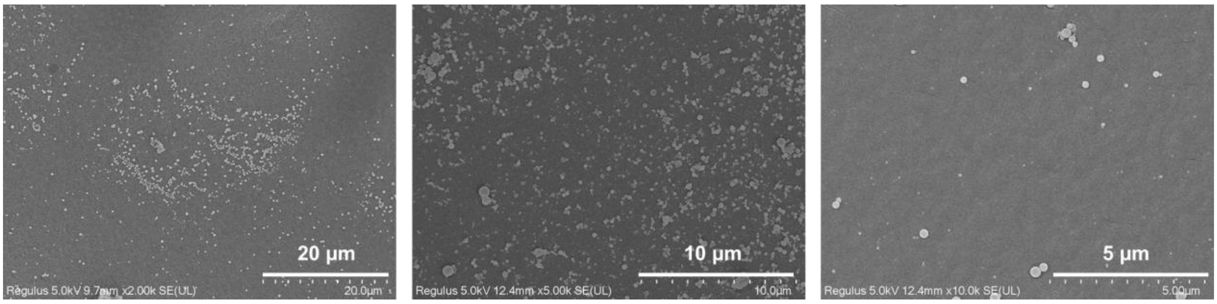

Figure S6. SEM images of sponge@MPN NPs ([TA] = 12 mg mL^{-1}) (obtained after incubation of sponge@TA-APTES with ferric sulfate).

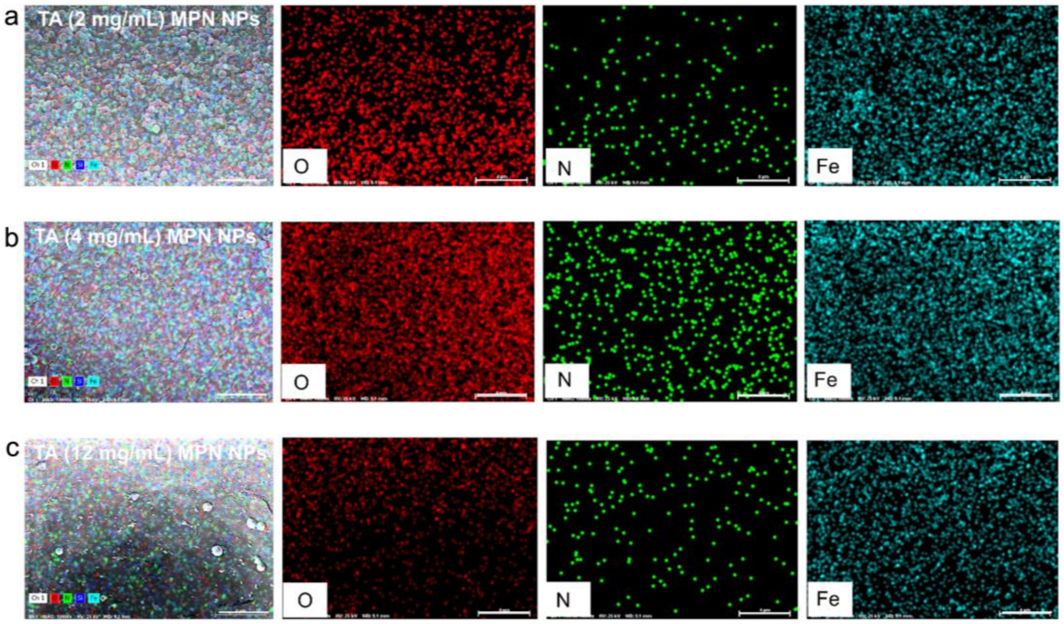

Figure S7. (a–c) EDX mapping of sponge@MPN NPs obtained using TA at varying concentrations of 2 mg mL^{-1} (a), 4 mg mL^{-1} (b), and 12 mg mL^{-1} (c).

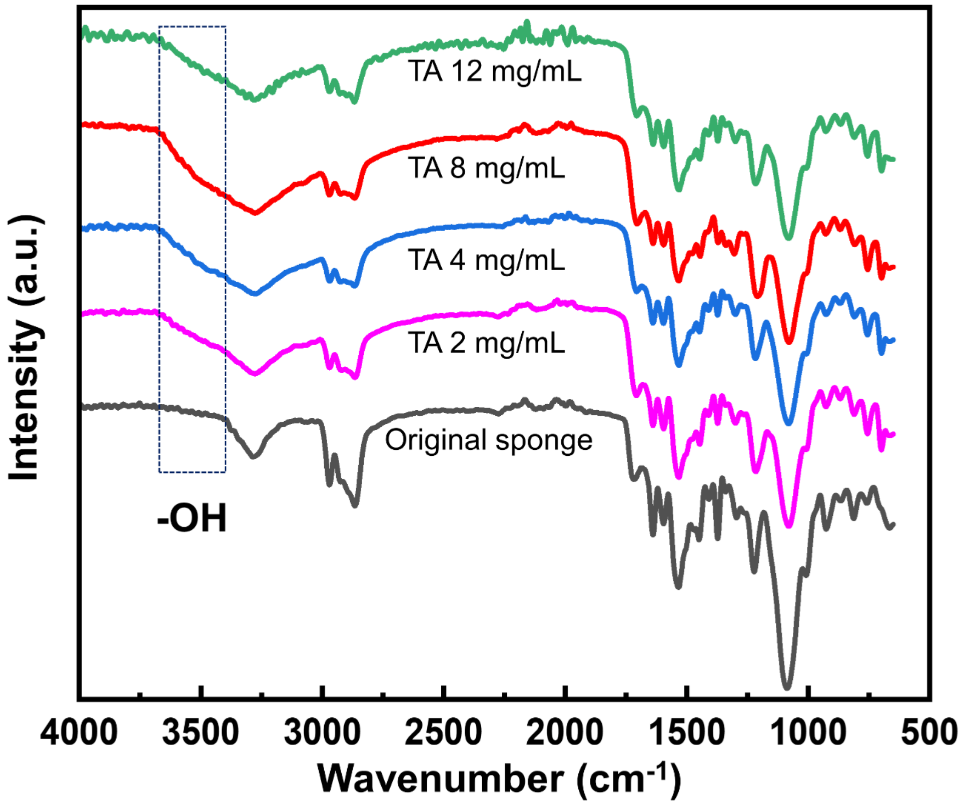

Figure S8. Attenuated total reflectance FTIR spectra of the original sponge and sponge@MPNs obtained using TA at varying concentrations.

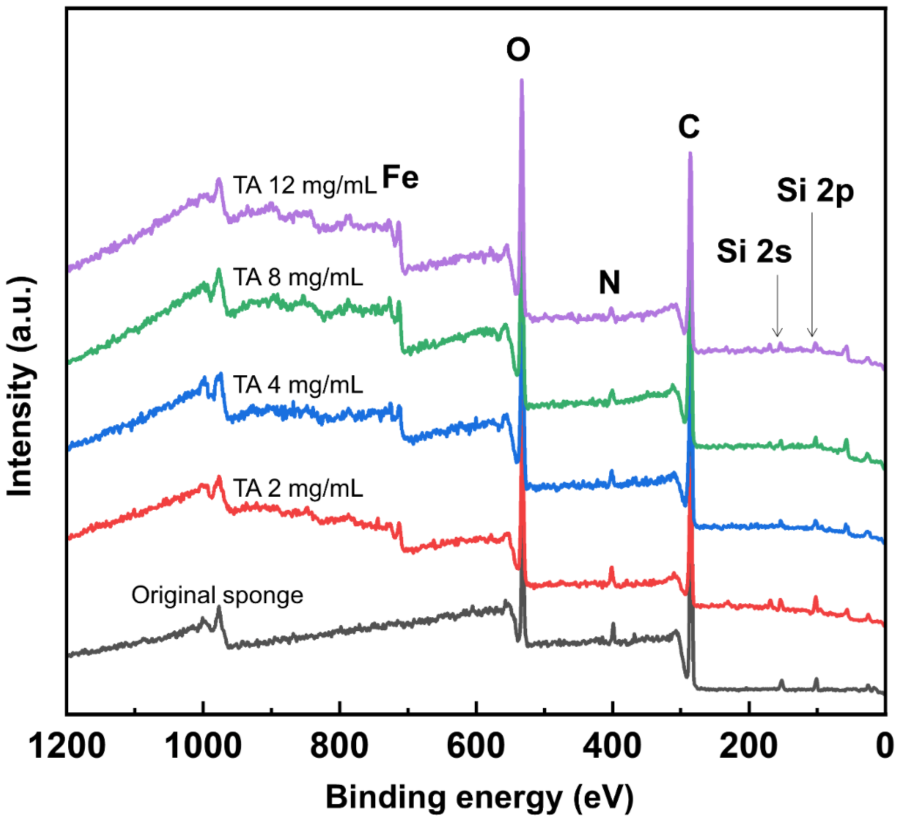

Figure S9. XPS spectra of the original sponge and different sponge@MPNs obtained using TA at varying concentrations.

Table S2. Elemental composition of different sponge@MPNs obtained using TA at varying concentrations

Sponge	Concentration of TA [mg mL^{-1}]	Composition [at.%]				
		C	N	O	Si	Fe
sponge@MPN NPs	2	58.91	3.70	30.43	5.84	1.11
sponge@MPN NPs	4	64.4	2.39	28.82	3.04	1.35
sponge@C-MPN	8	64.03	3.13	27.52	3.38	2.28
sponge@MPN NPs	12	63.92	2.26	29.36	2.92	1.60

Table S3. Peak areas and area ratios of C=O

Sponge	Concentration of TA [mg mL^{-1}]	Peak area		Area ratio C=O/Total
		C=O	Total	
sponge@MPN NPs	2	15024.2	26416.6	56.87%
sponge@MPN NPs	4	11703.6	27545.2	42.49%
sponge@C-MPN	8	10041.2	31477.5	32.89%
sponge@MPN NPs	12	11010.6	20483.7	53.75%

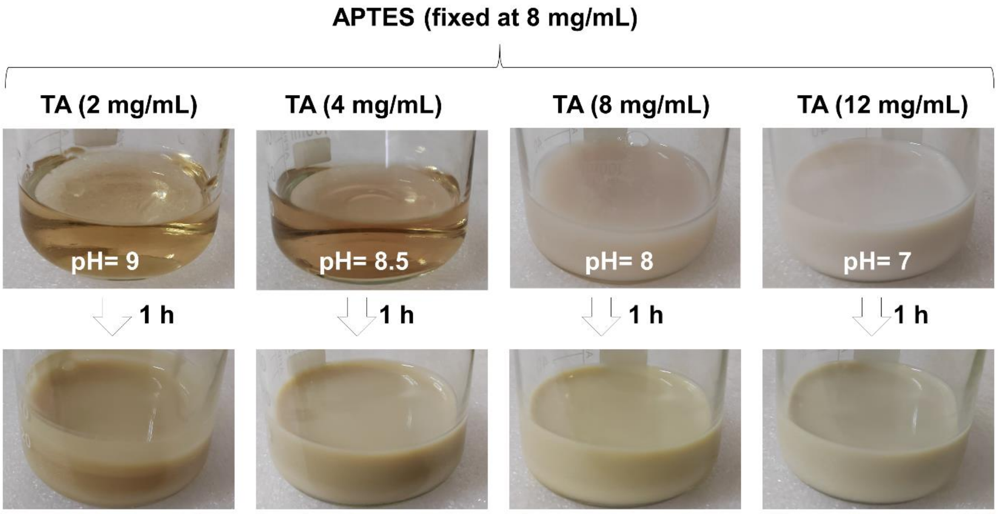

Figure S10. Photographs of TA/APTES reaction mixtures prepared with TA at different concentrations and their corresponding pH values.

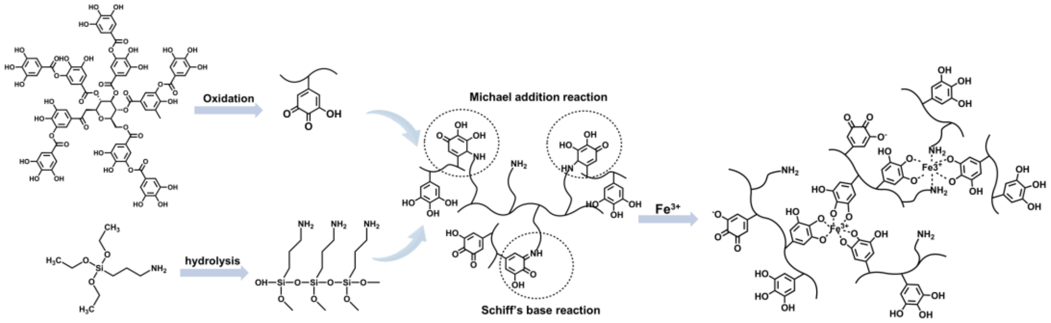

Figure S11. Schematic diagram illustrating the main reaction between TA and APTES and coordination interaction between Fe3+ and TA-APTES coating.

Table S4. Peak areas and area ratios of O–Fe

Sponge	Concentration of TA [mg mL^{-1}]	Peak area		Area ratio O–Fe/Total
		O–Fe	Total	
sponge@MPN NPs	2	4797.3	26416.6	18.16%
sponge@MPN NPs	4	4762.6	27545.2	17.29%
sponge@C-MPN	8	6100.3	31477.5	19.38%
sponge@MPN NPs	12	2988.57	20483.7	14.59%

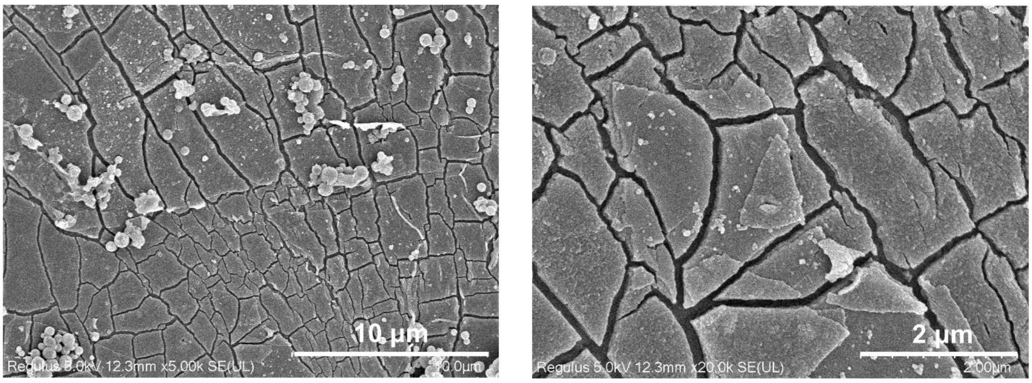

Figure S12. SEM images of sponge@C-MPN$_{30℃}$ at different magnifications.

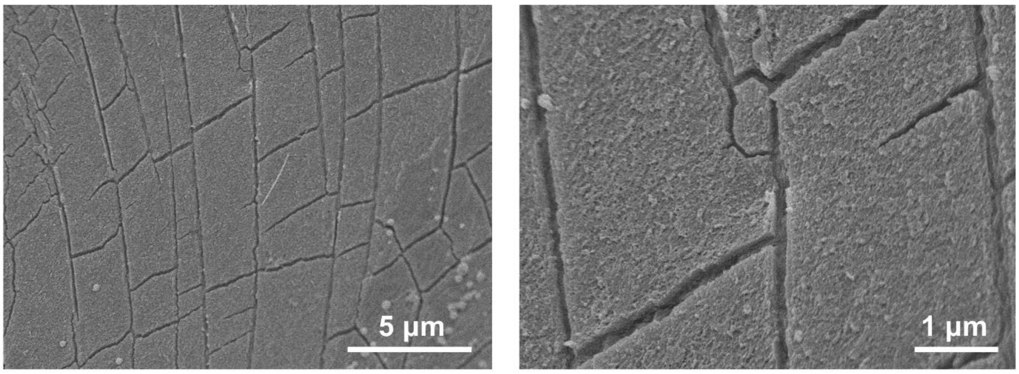

Figure S13. SEM images of sponge@C-MPN$_{60℃}$ at different magnifications.

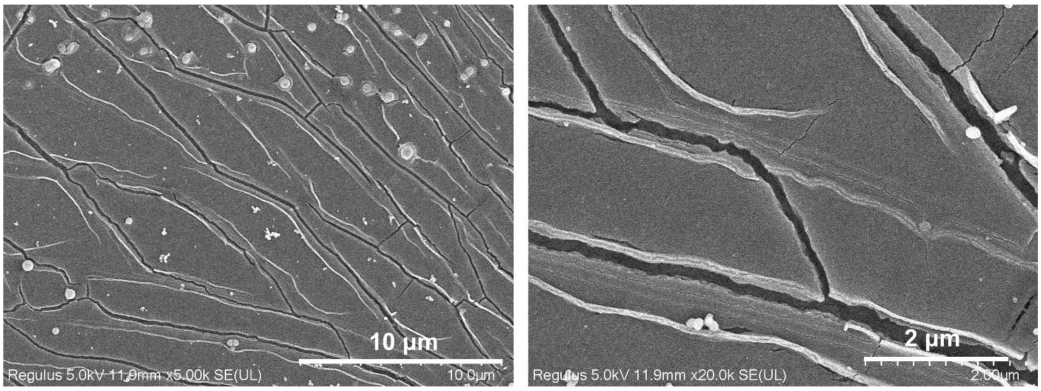

Figure S14. SEM images of sponge@C-MPN$_{80℃}$ at different magnifications.

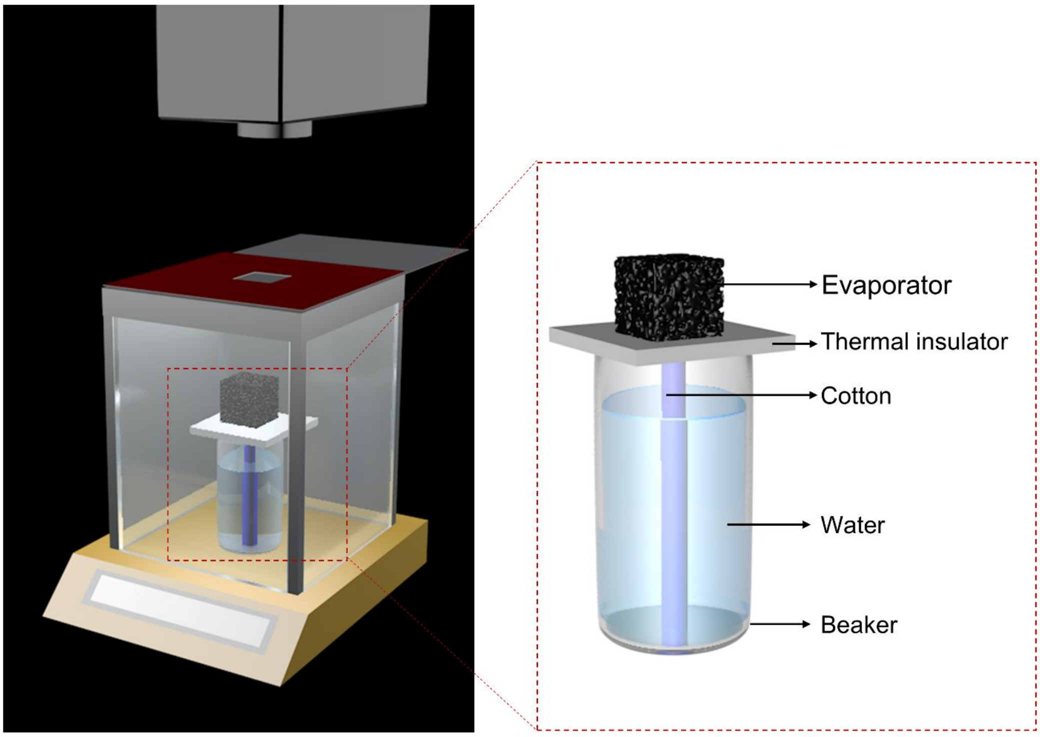

Figure S15. Schematic diagram of the equipment used for the solar evaporation studies.

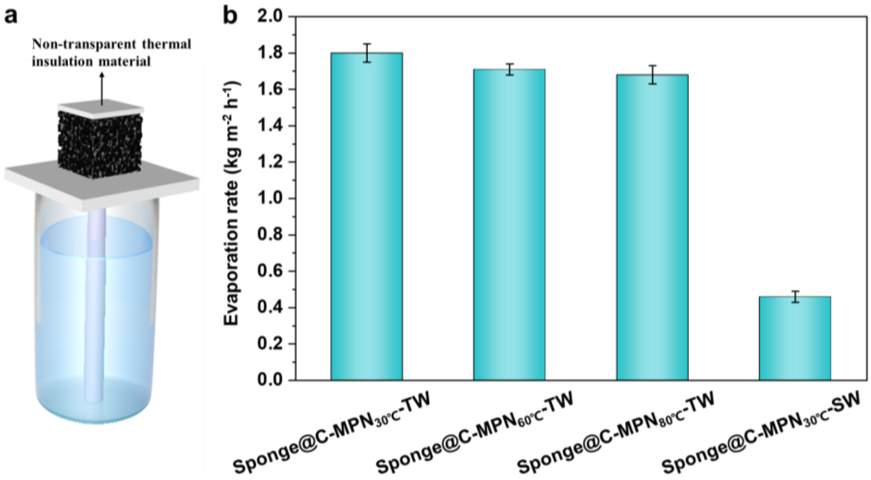

Figure S16. (a) Schematic of the experimental setup used to investigate the evaporation rate caused by side surface exposure under illumination. The top surface of the photothermal material was covered with a non-transparent thermal insulation material, whereas the four side surfaces remained uncovered. (b) Evaporation rates attributed to the side surfaces during illumination.

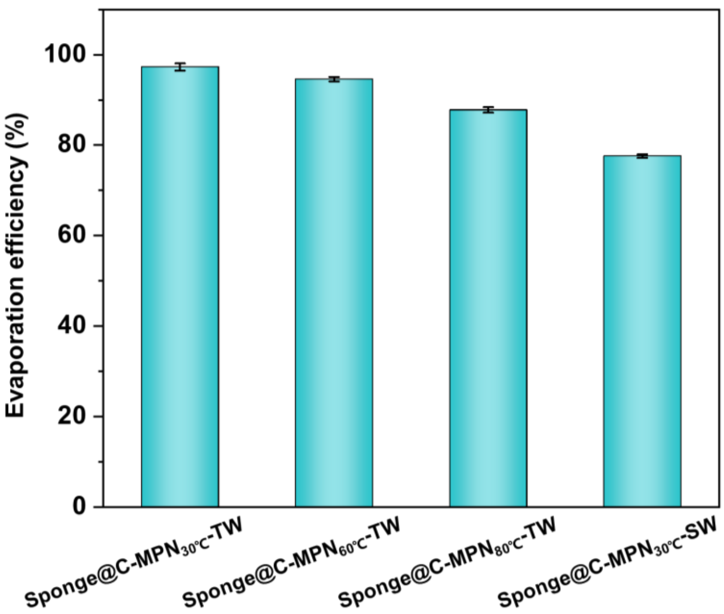

Figure S17. Evaporation efficiencies of different sponges.

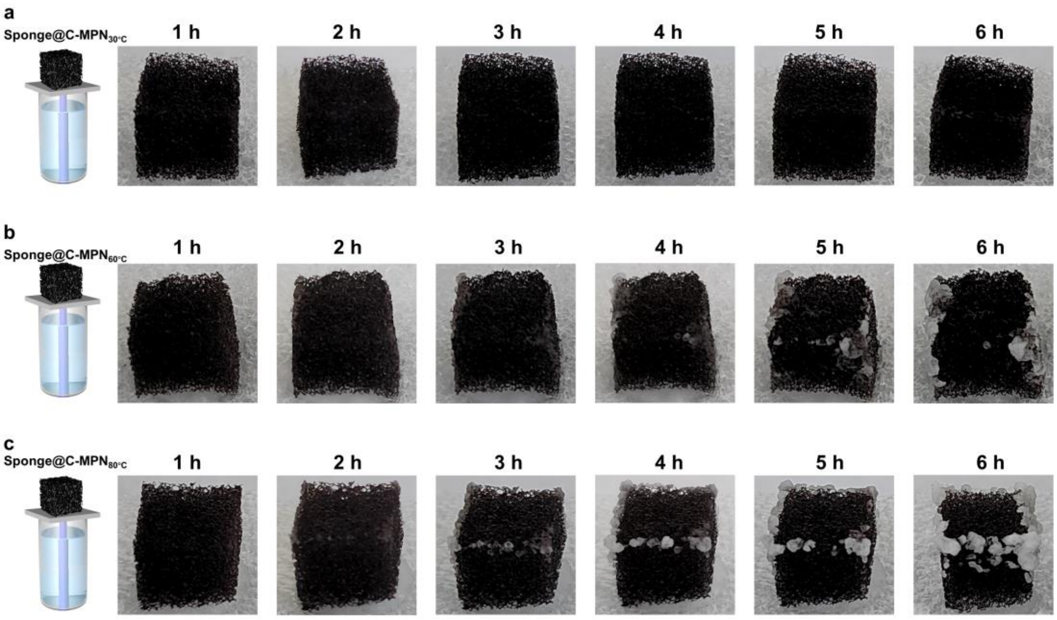

Figure S18. Photographs of (a) sponge@C-MPN$_{30°C}$, (b) sponge@C-MPN$_{60°C}$, and (c) sponge@C-MPN$_{80°C}$ used for the evaporation of 3.5% NaCl solution under 1 kW m^{-2} for different times.

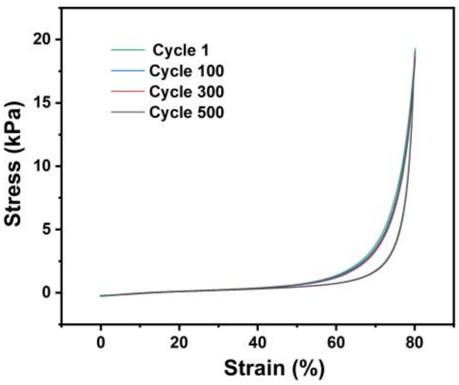

Figure S19. Stress–strain curves of sponge@C-MPN$_{30°C}$ under 80% strain across 500 cycles.

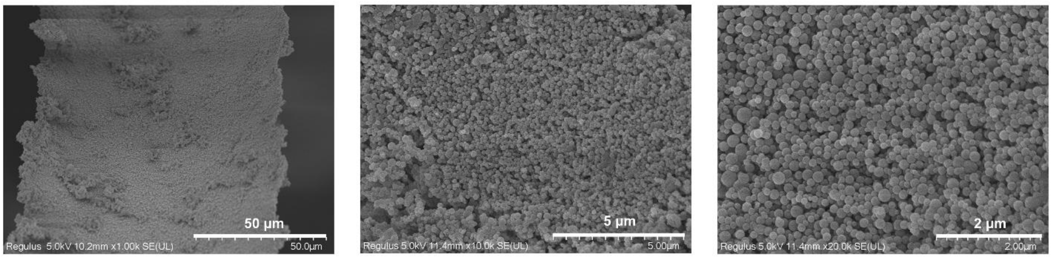

Figure S20. SEM images of sponge@S-MPNs at different magnifications before compression.

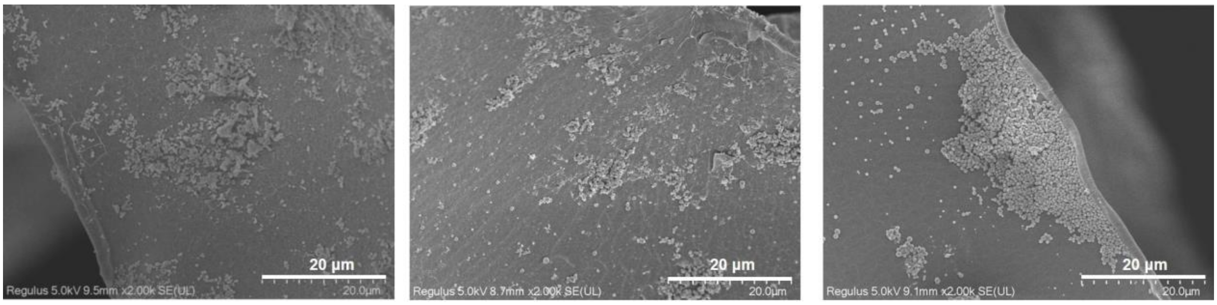

Figure S21. SEM images of sponge@S-MPNs after compression for 100 times.

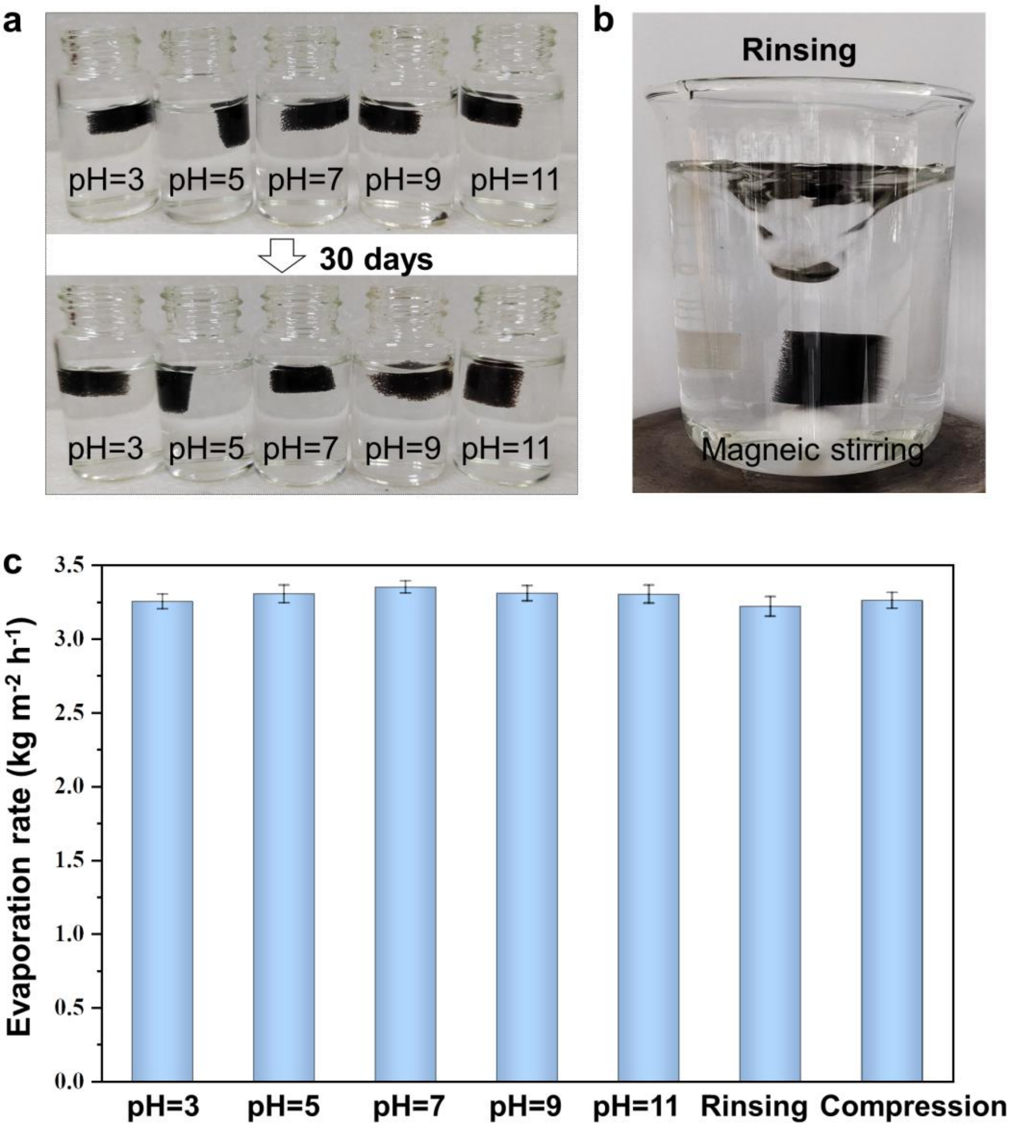

Figure S22. Stability of sponge@C-MPN$_{30°C}$. (a) Photographs of sponge@C-MPN$_{30°C}$ following immersion in water at different pH values for 30 days. (b) Photograph of sponge@C-MPN$_{30°C}$ subjected to vigorous rinsing (1000 rpm, 10 h daily, 30 days). (c) Water evaporation rate (under one sun irradiation) of sponge@C-MPN$_{30°C}$ subjected to different treatments (i.e., immersion in water at varying pH values for 30 days; vigorous and prolonged rinsing; compression). Data are shown as the mean ± standard deviation (SD, n = 4).

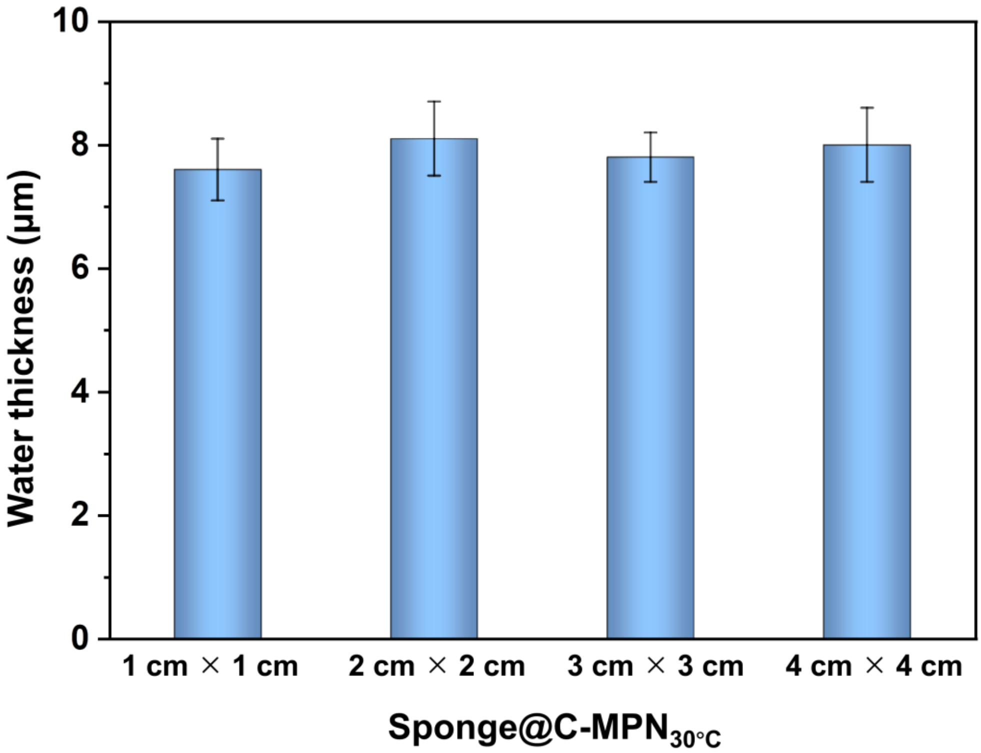

Figure S23. Water layer thickness of sponge@C-MPN$_{30°C}$ of varying dimensions. Data are shown as the mean ± SD (n = 4).

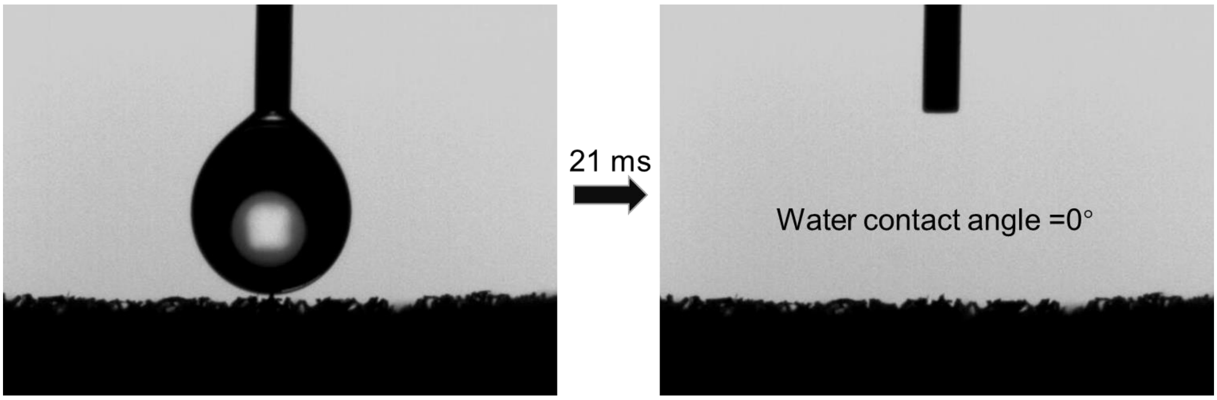

Figure S24. Photographs of the water contact angles of sponge@C-MPN$_{30°C}$ after 23 days of outdoor solar desalination.

Movie S1. Manual compression of the sponge for 300 cycles to demonstrate compressive durability.

Movie S2. Mechanical compression of the sponge for 500 cycles to demonstrate compressive resistance.

Movie S3. Water droplet behavior on sponge@C-MPN after 23 days of outdoor solar desalination.
